# Osmoregulatory contributions of the corticotropin-releasing factor system in the intestine of Atlantic salmon

**DOI:** 10.1242/jeb.250052

**Published:** 2025-05-15

**Authors:** Brett M. Culbert, Stephen D. McCormick, Nicholas J. Bernier

**Affiliations:** ^1^ Department of Integrative Biology, University of Guelph, 50 Stone Rd E, Guelph, Ontario, Canada N1G 2W1; ^2^ Department of Biology, University of Massachusetts, Amherst, Amherst, MA 01003, USA

**Keywords:** Chloride transport, Sodium transport, Seawater transfer, Smoltification, Urocortin, Water transport

## Abstract

The ability of euryhaline fishes to tolerate different environmental salinities depends upon the flexibility of their osmoregulatory organs, including the intestine. Several endocrine pathways contribute to the coordination of osmoregulatory processes in the teleost intestine; however, while the corticotropin-releasing factor (CRF) system has established osmoregulatory actions in the mammalian intestine, it is unclear whether the intestinal CRF system serves similar functions in teleosts. Therefore, we sought to determine whether the CRF system contributes to osmoregulatory processes in the intestine of Atlantic salmon (*Salmo salar*). We first showed using *in vitro* sac preparations that activation of CRF receptor type 2 (CRFR2) in the middle and posterior regions of the intestine reduces water, Na^+^ and Cl^−^ absorption. However, co-activation of CRFR1 and CRFR2 inhibited water and Na^+^ absorption without affecting net Cl^−^ absorption. We then assessed how the CRF system in the middle and posterior regions of the intestine was transcriptionally regulated during the seasonal acquisition of seawater tolerance (i.e. smoltification) and following changes in environmental salinity. Compared with parr, smolts had higher transcript levels of CRF ligands and this difference persisted following seawater transfer. Additionally, seawater transfer caused transient increases in transcription of urocortin 2 (*ucn2*) and *crfr2* (posterior intestine only). Similar increases in *ucn2* and *crfr2* mRNA were observed following seawater to freshwater transfer of post-smolts. Our results indicate that the intestinal CRF system of Atlantic salmon contributes to osmoregulation during the initial days following changes in environmental salinity and that osmoregulatory actions of the intestinal CRF system are conserved across vertebrates.

## Teaser

**Summary:** A combination of *in vivo* and *in vitro* studies reveals that the corticotropin-releasing factor system serves osmoregulatory functions in the intestine of Atlantic salmon.

## INTRODUCTION

The capacity for euryhaline fishes to survive in both fresh water (FW) and seawater (SW) relies on the flexibility of their osmoregulatory organs, including the gills, kidney and intestine (Evans et al., 2005; Grosell, 2011a; Larsen et al., 2014; Takvam et al., 2021). To survive in FW, fish actively absorb ions and minimize water intake, whereas SW-acclimated fish excrete excess ions and drink water (Takei et al., 2014). The intestine plays critical osmoregulatory roles in both environments. In FW, the intestine contributes to osmoregulation by absorbing ions that are primarily dietary in origin (Wood and Bucking, 2011). In contrast, the intestine of SW-acclimated fish processes the large amounts of SW that are ingested to overcome diffusive water loss in a hyperosmotic environment (up to 50-fold higher drinking rates than in FW; Grosell, 2011a). Specifically, ingested SW is partially desalinated in the esophagus, after which ions [both monovalent (Na^+^, K^+^ and Cl^−^) and divalent (Ca^2+^, Mg^2+^, SO_4_^2−^); transcellularly] and water (trans- and/or paracellularly) are absorbed across the length of the intestine (Grosell, 2011a). These excess ions are then excreted via the gills (monovalent) or kidney (divalent). However, while the osmoregulatory actions of the intestine in FW and SW are well established, the complete suite of hormones involved in regulating these functions (and the pathways mediating these effects) remain largely unclear.

Most studies investigating endocrine contributions to osmoregulation in teleosts have focused on the gills (McCormick, 2001; Takei et al., 2014), but many hormones also influence osmoregulatory processes in the intestine. This includes somatostatin peptides, natriuretic peptides, guanylin peptides and corticosteroids (Holmgren and Olsson, 2009; Takei and Loretz, 2011). Of these hormones, the contributions of cortisol have arguably been most thoroughly studied to date. Elevated cortisol production following changes in environmental salinity helps to coordinate the transport of ions and water across the intestinal epithelium by altering levels of several transporters and channels, including aquaporins, claudins and Na^+^-K^+^-ATPase (Breves et al., 2024; Choi et al., 2013; Sundell et al., 2003; Tipsmark et al., 2010; Veillette et al., 1995). Additionally, rates of cortisol synthesis in anadromous fishes increase prior to migrating, helping to alter osmoregulatory tissues (including the intestine) that are required for SW entry (McCormick, 2013). Increased cortisol synthesis during either of these processes is caused, at least in part, by increased activation of the central corticotropin-releasing factor (CRF) system (Choi et al., 2015; Craig et al., 2005; Culbert et al., 2022; Mousa and Mousa, 2006). Specifically, activation of CRF receptor 1 (CRFR1) on pituitary corticotropes causes the release of adrenocorticotropic hormone, which stimulates cortisol production by steroidogenic cells in the anterior kidney (Best et al., 2024). In this sense, the CRF system indirectly contributes to osmoregulatory processes in the teleost intestine by regulating cortisol synthesis. However, the CRF system also has direct osmoregulatory effects on peripheral tissues in other taxa. In mammals, activation of CRFR1 stimulates secretion of Cl^−^ and water across the intestine – especially the jejunum and colon (Rodiño-Janeiro et al., 2015; Stengel and Taché, 2009) – which is thought to be mediated by changes in components of tight junctions (Yu et al., 2013; Yue et al., 2017) and increased activity of basolateral Na^+^-K^+^-2Cl^−^ cotransporter type 1 (NKCC1; Liu et al., 2021). Similarly, diuretic hormone 44 (the insect analog of CRF; Cardoso et al., 2014; Coast, 2007) stimulates water, K^+^ and Na^+^ secretion across the Malpighian tubules of insects via interactions with NKCC1 and V-type H^+^-ATPase (Coast, 2007; Larsen et al., 2014). Yet, in spite of conserved osmoregulatory roles in mammals and insects, the relative contribution of the CRF system in coordinating osmoregulatory processes in fish remains unclear.

The teleost CRF system consists of five ligands [CRFa, CRFb, urotensin 1 (UTS1), urocortin 2 (UCN2) and UCN3], two receptors (CRFR1 and CRFR2) and a binding protein (CRFBP; Best et al., 2024; Maugars et al., 2022). A series of pioneering studies reported that UTS1 can directly contribute to osmoregulatory processes across a variety of peripheral tissues of fish (Chan, 1975; Loretz et al., 1981; Mainoya and Bern, 1982; Marshall and Bern, 1979, 1981). However, despite significant advancements in our understanding of the CRF system and osmoregulatory processes since this period, few studies have followed up on this work. Indeed, the effect of osmotic disturbances on transcript levels of CRF system components in peripheral tissues has only been evaluated in a handful of studies, with all having evaluated responses in the gills (Aruna et al., 2012, 2021; Culbert et al., 2025a). Additionally, it is unclear whether these effects are mediated by locally produced CRF ligands (i.e. auto- and/paracrine regulation), ligands delivered via the circulation (i.e. endocrine regulation) or a combination of both. Consequently, our understanding of interactions between osmoregulation and the CRF system in peripheral tissues of fish remains limited. This includes the intestine, with only a single study reporting that *in vitro* treatment of intestinal sacs with UTS1 reduced absorption of Na^+^, Cl^−^ and water in Mozambique tilapia (*Oreochromis mozambicus*; Mainoya and Bern, 1982). As such, it remains unclear whether the CRF system has similar functions in the intestine of other teleosts, and it is still unknown whether the osmoregulatory actions associated with CRF system activity in the teleost intestine are mediated by CRFR1 or CRFR2. Therefore, the objective of the current study was to determine whether the CRF system also serves osmoregulatory functions in Atlantic salmon (*Salmo salar*), and which receptor(s) mediate these effects.

Working under the hypothesis that the CRF system has evolutionarily conserved osmoregulatory actions, we initially set out to determine whether activation of the intestine CRF system would reduce water and ion absorption across the intestine of Atlantic salmon. Specifically, we predicted that, as in mammals (Rodiño-Janeiro et al., 2015; Stengel and Taché, 2009), reductions in intestinal ion and water absorption would be mediated by activation of CRFR1. To test this prediction, we pharmacologically manipulated activity of the CRF system using *in vitro* intestinal sacs and evaluated how these manipulations affected rates of Na^+^, Cl^−^ and water transport. We then determined which components of the CRF system were most transcriptionally abundant in different regions of the intestine and assessed how the major components changed: (1) during the seasonal acquisition of SW tolerance (i.e. smoltification); (2) following FW–SW transfer; and (3) following SW–FW transfer. We predicted that levels of CRF system components would decrease during the parr-to-smolt transformation and following FW–SW transfer (allowing for high rates of water absorption) but would increase following transfer from SW–FW (contributing to reductions in water absorption). Collectively, our study provides the most comprehensive evaluation of potential osmoregulatory functions of the intestinal CRF system that has been conducted in any non-mammalian vertebrate.

## MATERIALS AND METHODS

### Experimental animals and housing

Experiments 1 and 3 used sexually immature Atlantic salmon that were acquired from the Normandale Fish Culture Station (Vittoria, ON, Canada) and held in the Hagen Aqualab at the University of Guelph (Guelph, ON, Canada). Fish originated from a landlocked source population (Sebago Lake, Maine, USA), which have been bred in captivity since 1999. All fish were maintained in 1.8 m diameter fibreglass tanks (∼2000 liters) that were supplied with aerated, flow-through well water (<1 ppt) maintained at 12°C and were kept on a 12 h light:12 h dark photoperiod regime. Fish were fed to satiation three times per week with commercial pellets (Blue Water Fish Food, Guelph, ON, Canada). A stocking density of ∼100 fish per tank was maintained and fish were kept under these conditions for several months prior to starting experiments. All procedures were carried out in accordance with the Canadian Council on Animal Care guidelines for the use of animals in research and teaching and were approved by the University of Guelph's Animal Care Committee (AUP #4123).

Experiment 2 took place at the US Geological Survey (USGS) S.O. Conte Anadromous Fish Research Laboratory (Turners Falls, MA, USA) using sexually immature Atlantic salmon parr that were obtained from the Kensington State Hatchery (Kensington, CT, USA) in the autumn of 2018. Fish were held in 1.8 m diameter tanks that were supplied with flow-through ambient Connecticut River water (<1 ppt) at a flow rate of 4 liters min^−1^ and tanks were continuously aerated. Tanks were indoors, but experienced the natural photoperiod, and fish were fed to satiation (BioOregon, Westbrook, ME, USA) using automatic feeders. In December of 2018, fish were separated by size into parr and pre-smolt groups as described previously (Culbert et al., 2022). Each group of fish was maintained in duplicate tanks containing ∼100 fish and all fish experienced identical temperature regimes throughout the experiment (see details of experiment 2 below for more information). All fish rearing and sampling protocols were carried out in accordance with USGS institutional guidelines and protocol LSC-9096 that was approved by the USGS Eastern Ecological Science Center Institutional Animal Care and Use Committee.

### Experiment 1: *In vitro* evaluation of osmoregulatory contributions of the CRF system in the intestine

To evaluate whether the CRF system might contribute to the rate at which water, Na^+^ and/or Cl^−^ are transported across the intestine, *in vitro* intestinal sacs were prepared similarly to previous salmonid studies (e.g. Collie and Bern, 1982; Genz et al., 2011; Sundell et al., 2003; Veillette et al., 1995). After fasting for 48 h, juvenile Atlantic salmon parr [*N*=33; fork length (FL): 18.7±0.2 cm; mass: 68.2±2.2 g; mean±s.e.m.] were euthanized via cerebral concussion followed by spinal severance and the entire intestine posterior to the cecae was collected. Based on the description provided in Sundh et al. (2014), this included the middle (M. Int; between the final pyloric caeca and the ileorectal sphincter) and the posterior (P. Int; posterior to the ileorectal sphincter) regions, but not the anterior region (portion with caeca). Following removal of attached fat and vasculature, a heat-flared cannula (PE60, INTRAMEDIC; Becton Dickinson, Franklin Lakes, NJ, USA) was inserted in the anterior end of the intestine and attached using a polyamide suture (4-0 SUPRAMID EXTRA; S. Jackson, Inc., Alexandria, VA, USA). The intestine was then flushed five times with 1 ml of a modified Cortland's saline (NaCl: 124 mmol l^−1^, CaCl_2_: 1.6 mmol l^−1^, KCl: 5.1 mmol l^−1^, NaH_2_PO_4_: 3.0 mmol l^−1^, NaHCO_3_: 11.9 mmol l^−1^, MgSO_4_: 0.9 mmol l^−1^, glucose: 5.6 mmol l^−1^, HEPES: 5 mmol l^−1^; Wolf, 1963). All saline was brought to 12°C and gassed with 0.3% CO_2_ in O_2_ for an hour prior to the start of experiments. Additionally, the pH was adjusted to 7.8 using NaOH immediately prior to the start of these experiments. Preliminary experiments showed that pH of the serosal saline was stable for the duration of the experiments. Once cleared of its contents, a suture was tied on the posterior end of the intestine, and the sac was filled with saline containing either vehicle (0.1% DMSO), CRFa (1 µmol l^−1^ CRFa2 with 0.1% DMSO) or UCN2 (1 µmol l^−1^ UCN2b with 0.1% DMSO). In teleosts, CRFa activates both CRFR1 and CRFR2, while UCN2 is a specific agonist of CRFR2 (Hosono et al., 2015; Manuel et al., 2014). Both peptides were custom synthesized by GenScript Biotech (Piscataway, NJ, USA) according to the deduced amino acid sequences for Atlantic salmon on GenBank (see Table S1). As in previous studies (e.g. Genz et al., 2011; Ruhr et al., 2016; Whittamore et al., 2016), mannitol was used to maintain an equal osmolality across all solutions. The osmolality of these solutions (294±2 mOsm kg^−1^) was determined using a vapour pressure osmometer (Vapro 5520, Wescor) and was comparable with plasma osmolality values for Atlantic salmon (e.g. Culbert et al., 2025a). Furthermore, these values were consistent across treatment groups. To obtain a pre-incubation luminal saline sample, saline in the filling syringe was flushed back and forth between the sac and the syringe three times and the remaining saline was kept. The filling cannula was then sealed, and the weight of the sac was recorded. To determine how much saline was added to each sac, the filling syringe was weighed before and after filling. The sac was then placed into 15 ml of saline inside of a glass scintillation vial for 2 h. Symmetrical conditions were used to better facilitate comparisons with the results of Mainoya and Bern (1982). During this period, temperature was maintained at 12°C using a water bath and the serosal saline was continuously gassed with 0.3% CO_2_ in O_2_. After 2 h, sacs were removed, blotted dry, weighed, and a luminal saline sample was collected using the catheter. Following this, the catheter and sutures were removed, the sac was opened and an image was taken to determine surface area using ImageJ (v. 1.54j; Schneider et al., 2012).

A follow-up experiment to examine the specific contribution(s) by each CRFR was conducted using similar methods. The only difference was that sacs from juvenile salmon parr (*N*=34; FL: 18.5±0.3 cm, mass: 67.8±2.9 g) were filled with saline containing either vehicle (0.1% DMSO), CRFa (1 µmol l^−1^ CRFa2 with 0.1% DMSO), or CRFa and the CRFR1 antagonist antalarmin (CRFa+ANT; 1 µmol l^−1^ CRFa2 and 10 µmol l^−1^ ANT with 0.1% DMSO). Previous studies have shown that this antagonist is specific for CRFR1 in teleosts (Alderman et al., 2018; Culbert et al., 2025a; Lastein et al., 2008; Medeiros et al., 2014; Williams and Bernier, 2020). Antalarmin hydrochloride was purchased from Cayman Chemical (Product #15147; Ann Arbor, MI, USA).

Water flux was calculated as the difference in mass of the sac before and after the 2 h flux period and was adjusted both for tissue surface area (cm^2^) and time (h). Similarly, ion flux was calculated as the difference between the initial and final [Na^+^] and [Cl^−^] in the luminal saline per cm^2^ of tissue per hour. [Na^+^] was determined using a Jenway PFP7 flame photometer (Cole-Parmer, Vernon Hills, IL, USA) and [Cl^−^] was determined using a colorimetric assay (Zall et al., 1956). Standard curves (range of 0–200 µmol l^−1^ for Cl^−^ and 0–217 µmol l^−1^ for Na^+^) were made via dilution of a standardized NaCl solution (cat. no. 035616.AP; Thermo Fisher Scientific, Mississauga, ON, Canada), and all samples were diluted 1000-fold prior to measurement. Analyses for each experiment were conducted in a single assay and intra-assay variation [% coefficient of variation (% CV)] was 8.6% and 3.1% for the Na^+^ and Cl^−^ assays, respectively.

### Experiment 2: Regulation of the intestine CRF system during smoltification and FW–SW transfer

Parr (*N*=84; FL: 11.1±0.1 cm; mass: 14.0±0.38 g) and smolts (*N*=84; FL: 16.5±0.1 cm; mass: 44.8±1.0 g) were sampled on 19 February, 1 April, 6 May and 15 July, 2019 (*N*=12 per group per time point). Fish were kept at ambient temperatures (2–4°C) through the winter and water temperature was increased by 1°C per day to 8–9°C beginning on 15 February. This temperature was maintained throughout the spring so that all sampling points would be at identical temperatures. After 30 May, fish experienced normal summer temperatures (maximum of 18.4°C) until water temperature was decreased again (by 1°C per day to 9–10°C) on 3 July. To account for potential tank effects, fish from each group (parr or smolts) were sampled from two replicate tanks at each time point (*N*=6 per tank). To determine the response of fish following SW exposure, groups of parr and smolts were placed into six 1 m diameter tanks (3 tanks of parr and 3 tanks of smolts; *N*=12 per tank) containing 28 ppt recirculating SW (Instant Ocean Sea Salt, Blacksburg, VA, USA) during the week of 6 May, 2019. We used this concentration of SW to ensure 100% survival in both groups since parr are unable to tolerate full strength SW (35 ppt) for more than a few days (McCormick et al., 2013). These tanks were held at 8.5–9.5°C and contained particle, biological and charcoal filtration, as well as continuous aeration. Fish were fed to satiation every day, but food was withheld the day prior to samplings. Tanks of parr and smolts were sampled after either 24, 96 or 240 h of SW exposure. All fish were terminally anesthetized using NaHCO_3_ (12 mmol l^−1^) buffered MS-222 (100 mg l^−1^; pH 7.0) after which FL and mass were recorded. Blood was collected from the caudal vasculature using a 1 ml ammonium heparinized syringe, spun at 3200 ***g*** for 5 min at 4°C, and plasma was collected for later measurement of cortisol and osmolality. The intestinal tract was removed from each fish, flushed of its contents, regionally dissected according to Sundh et al. (2014), and the M. Int and P. Int regions were collected. All samples were immediately flash frozen in dry ice prior to being stored at −80°C for later RNA extraction and quantitative polymerase chain reaction (qPCR).

As an initial determination of which components of the CRF system were most abundant in each region of the intestine, we conducted qPCR on three separate pools of cDNA that each contained equal amounts of cDNA originating from a parr and a smolt that were sampled in February. Values for each transcript were corrected for primer efficiency (see below) and were expressed relative to the abundance of *crfbp1* in the posterior intestine, which was the CRF system component with the lowest levels that consistently amplified. Note that Atlantic salmon have two paralogs of each component of the CRF system owing to the salmonid-specific genome duplication (Lien et al., 2016). Therefore, we measured the individual paralogs of all CRF system components, except for UCN3 which shares 98% identity across paralogs.

### Experiment 3: Regulation of the intestine CRF system during SW–FW transfer

Post-smolt Atlantic salmon (*N*=28; FL: 37.8±0.5 cm; mass: 537.8±24.0 g) were held in a recirculating tank containing ∼2000 liters of SW (33 ppt; Instant Ocean Sea Salt) for approximately 6 months. This tank was continuously aerated with an air stone and was equipped with both particle and biological filtration, as well as UV sterilization. At the start of the experiment, *N*=10 salmon were immediately sampled while the remaining fish were split among two ∼500 liter tanks containing aerated, flow-through well water (<1 ppt). Either 24 or 96 h post-transfer, fish were euthanized using 0.2% phenoxyethanol (Sigma-Aldrich, St Louis, MI, USA), and their plasma (for cortisol and osmolality) and intestine segments (for qPCR) were collected as described above. We also sampled FW-acclimated salmon (*N*=10; FL: 39.4±1.0 cm; mass: 581.3±42.3 g) that had never been transferred to SW for comparison (see ‘Experimental animals and housing’ for housing conditions).

### Plasma cortisol and osmolality

Circulating cortisol levels were determined in duplicate using a previously validated direct competitive enzyme immunoassay (EIA; Carey and McCormick, 1998) in experiment 1, or a commercially available EIA (Neogen, Lexington, KY, USA; cat. no. 402710) for all other experiments. Intra- and inter-assay variation for cortisol measurement was 9.4% and 12.4% CV, respectively. Plasma osmolality values were determined in duplicate using a vapour pressure osmometer (Vapro 5520, Logan, UT, USA) and had an intra-assay variation of 8.7% CV.

### RNA isolation and qPCR

Intestinal segments were first ground on dry ice using a mortar and pestle and were then homogenized in TRIzol reagent (Invitrogen) using a Precellys Evolution tissue homogenizer (Bertin Instruments, Montigny-le-Bretonneux, France). Following the manufacturer's protocol, total RNA was extracted, and its quantity and purity were assessed using a NanoDrop 2000 spectrophotometer (Thermo Fisher Scientific). Following this, we treated 1 µg of RNA with DNase (DNase 1; Thermo Fisher Scientific) and reverse transcribed cDNA using a high-capacity cDNA reverse transcription kit (Applied Biosystems, Waltham, MA, USA). We then performed qPCR using a CFX96 system (Bio-Rad, Hercules, CA, USA) with SYBR green (SsoAdvanced Universal; Bio-Rad) and all samples were run in duplicate. Negative controls, including no template controls (where cDNA was replaced with water) and no reverse transcriptase controls (where reverse transcriptase was replaced with water during cDNA synthesis) were also included. Each reaction contained a total of 20 µl, which consisted of 10 µl SYBR green, 5 µl combined forward and reverse primers (0.2 µmol l^−1^ [final]), and 5 µl of 10× diluted cDNA. Cycling parameters included a 30 s activation step at 95°C, followed by 40 cycles consisting of a 3 s denaturation step at 95°C and a combined 30 s annealing and extension step at 60°C. Melting curve analysis was conducted at the end of each run to confirm the specificity of each reaction. To account for differences in amplification efficiency, standard curves were constructed for each gene using serial dilutions (4×) of pooled cDNA. Input values for each gene were obtained by fitting the average threshold cycle value to the antilog of the gene-specific standard curve, thereby correcting for differences in primer amplification efficiency. To correct for minor variations in template input and transcriptional efficiency, we normalized our data to the geometric mean of transcript abundances of elongation factor 1α (*ef1α*) and ribosomal protein L13a (*rpl13a*) as reference genes. Reference gene levels were stable across treatment groups for all experiments and all data are expressed relative to the mean value of the control group within each experiment (see figure captions for further details).

### Statistical analyses

Statistical analyses were performed using R (v4.4.0; r-project.org) and graphs were created using GraphPad Prism (v. 10.4.1; GraphPad Software Inc.). All data are presented as means±1 standard error of the mean (s.e.m.) and a significance level (α) of 0.05 was used for all tests. Outliers were excluded based on a 2× interquartile range threshold. When data did not meet the assumptions of normality and/or equal variance, data were either log or square-root transformed to improve the fit of the model. Data for experiments 1 and 3 were analyzed using one-way ANOVAs that contained either treatment (experiment 1) or time (experiment 3) as a factor. Data for experiment 2 were analyzed using two-way ANOVAs that included group (parr and smolt) and either month (February, April, May and July) or time following SW exposure (FW, 24, 96 and 240 h) as factors, as well as the interaction between these factors. When significant differences were detected, *post hoc* Tukey's tests were performed using the ‘emmeans’ package (https://CRAN.R-project.org/package=emmeans).

## RESULTS

### *In vitro* effects of CRF system activity on osmoregulatory processes in the intestine

**Fig. 1.**
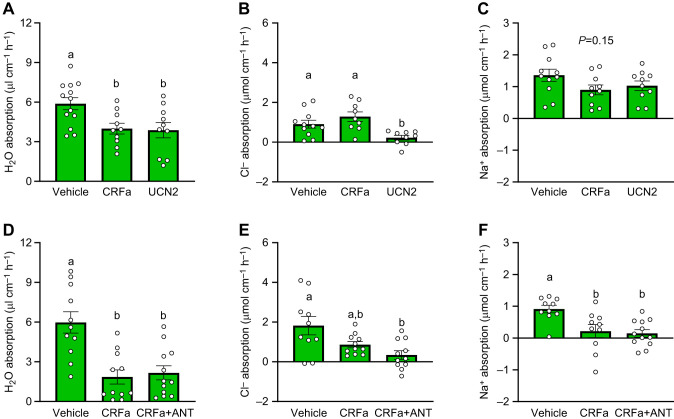
**Effects of corticotropin-releasing factor (CRF) system activity on intestinal osmoregulatory processes.** Changes in rates of water (H_2_O; A,D), chloride (Cl^−^; B,E) and sodium (Na^+^; C,F) absorption across the combined middle and posterior intestinal segments of Atlantic salmon (*Salmo salar*) parr. (A–C) In experiment 1, intestines were treated with vehicle, corticotropin-releasing factor a2 (CRFa) or urocortin 2b (UCN2). (D–F) In experiment 2, intestines were treated with vehicle, CRFa or CRFa plus the CRF receptor 1-specific antagonist antalarmin (CRFa+ANT). Significant differences (*P*<0.05; one-way ANOVA) are depicted using letters. Values are represented as means±s.e.m. and individual data points are shown (*N*=9–13).

Rates of water absorption were significantly affected by treatment with CRF peptides (Fig. 1A; *P*=0.008). Specifically, intestines that were treated with either CRFa (*P*=0.03) or UCN2 (*P*=0.02) absorbed ∼35% less water compared with vehicle-treated control intestines, but no difference was detected when comparing water absorption rates of CRFa- versus UCN2-treated intestines (*P*=0.99). In contrast, while rates of Cl^−^ absorption (Fig. 1B; *P*=0.004) did not differ between CRFa and control intestines (*P*=0.38), Cl^−^ uptake was reduced by 75–85% following UCN2 treatment compared with either control (*P*=0.046) or CRFa (*P*=0.003). Rates of Na^+^ absorption were not significantly affected by treatment with CRF peptides (Fig. 1C; *P*=0.15) but tended to be ∼30% lower in both treatment groups compared with control intestines.

The inhibitory effect of CRFa on water absorption was not affected by blocking CRFR1 with its antagonist antalarmin (Fig. 1D; *P*<0.001) since rates of water absorption did not differ between CRFa and CRFa+ANT groups (*P*=0.94), but rates in both groups were ∼60% lower than the control group (both *P*<0.001). In contrast, rates of Cl^−^ absorption (Fig. 1E; *P*=0.01) in the CRFa group were not different from the control (*P*=0.40) or CRFa+ANT groups (*P*=0.13), but the CRFa+ANT group absorbed ∼80% less Cl^−^ than the control group (*P*=0.007). Rates of Na^+^ absorption (Fig. 1F; *P*=0.002) were ∼80% lower in intestines treated with CRFa (*P*=0.009) or CRFa+ANT (*P*=0.003) compared with the vehicle control. No difference in Na^+^ absorption was observed between CRFa and CRFa+ANT groups (*P*=0.94).

### Abundance of CRF system components in the intestine

**Fig. 2.**
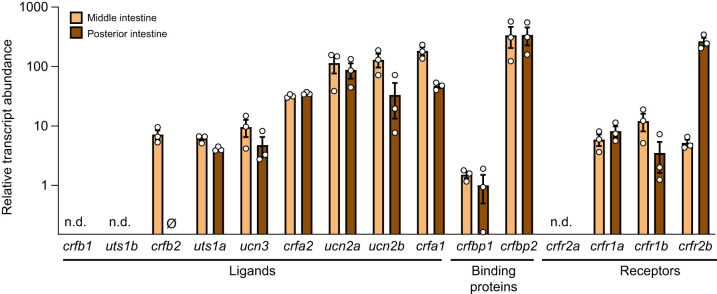
**Characterization of the intestinal CRF system.** Relative abundance of individual ligands, binding proteins and receptors of the corticotropin-releasing factor system in the middle and posterior regions of the intestine in Atlantic salmon. Bars represent mean abundance (±s.e.m.) of each component relative to *crfbp1* in the posterior intestine (the component with the lowest detectable levels). Each point (*N*=3) represents a pooled sample collected from a parr and a smolt sampled in February. Components that were not detected in either tissue are indicated with n.d. (not detected) and the ∅︀ symbol indicates instances where components were not detectable in one of the regions. Note that data are plotted on a log_10_ scale for visualization purposes.

Most components of the CRF system were reliably detected in both regions of the intestine (Fig. 2) and the abundance of these components was either similar between regions or higher in the middle relative to the posterior intestine (except for *crfr2b*; see below). All CRF ligands were detectable except for *crfb1* (undetectable in both regions), *uts1b* (undetectable in both regions) and *crfb2* (undetectable in posterior intestine). Of the CRF ligands which were detected, *crfa1*, *ucn2b*, *ucn2a* and *crfa2* displayed the highest levels and were ∼10- to 20-fold more abundant than *crfb2* (in middle intestine only), *uts1a* and *ucn3*. Both CRF binding proteins were detectable, but levels of *crfbp2* – the component with the greatest abundance (amplified at ∼26.5 cycles) – were ∼300× greater than *crfbp1* (the component with the lowest detectable levels). Finally, all CRF receptors were detectable except for *crfr2a* (both regions). However, while levels of *crfr1a*, *crfr1b* and *crfr2b* (in the middle intestine) tended to be low, levels of *crfr2b* in the posterior intestine (∼40–50× greater than all other receptors) were the second most abundant CRF system component overall (amplified at ∼27.5 cycles).

### Seasonal changes in the intestine CRF system (parr and smolts)

**Fig. 3.**
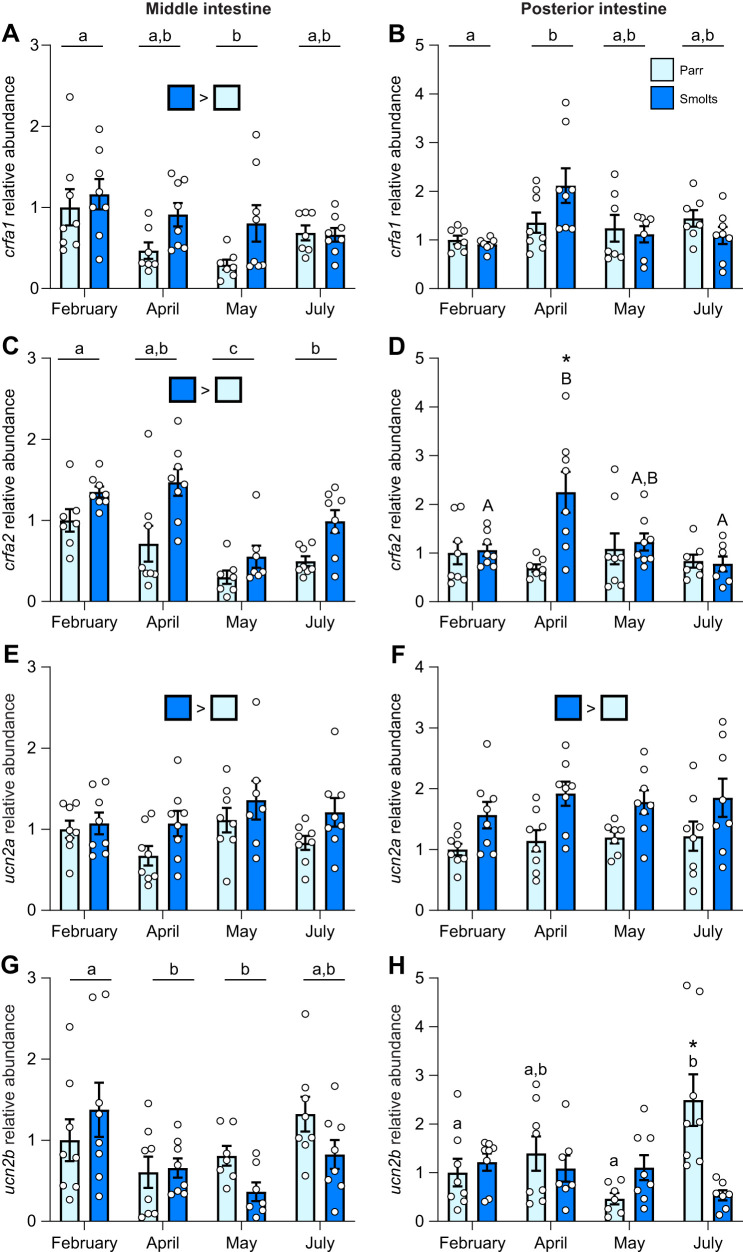
**Changes in intestinal CRF ligands during smoltification.** Seasonal changes in transcript abundance of corticotropin-releasing factor a1 (*crfa1*; A,B), a2 (*crfa2*; C,D), urocortin 2a (*ucn2a*; E,F) and 2b (*ucn2b*; G,H) in the middle (left) or posterior (right) region of the intestine in pre-migratory parr and migratory smolt Atlantic salmon. Significant differences (*P*<0.05; two-way ANOVA) are depicted using either letters (across time; uppercase=within smolts, lowercase=within parr; underlined=overall time effect), filled oversized squares (between groups across all time points) or asterisks (between groups within a time point). Data are expressed relative to parr in February and have been normalized to the geometric mean of *ef1a* and *rpl13a*. Values are represented as means±s.e.m. and individual data points are shown (*N*=5–8).

Levels of *crfa1* in the middle intestine (Fig. 3A; *P*_group_=0.004, *P*_time_<0.001, *P*_group×time_=0.09) were ∼30% higher in smolts versus parr across all time points, and averaged values combining both groups were ∼50% lower in May than February. In the posterior intestine, levels of *crfa1* (Fig. 3B; *P*_group_=0.91, *P*_time_=0.02, *P*_group×time_=0.10) were not different between parr and smolts but were ∼2-fold higher in April than February when comparing averaged values combining both groups. Similarly to *crfa1*, levels of *crfa2* in the middle intestine (Fig. 3C; *P*_group_<0.001, *P*_time_<0.001, *P*_group×time_=0.38) were ∼75% higher in smolts versus parr across all time points and were lowest in May when comparing averaged values combining both groups. Levels of *crfa2* were also high in the posterior intestine of smolts (Fig. 3D; *P*_group_=0.01, *P*_time_=0.11, *P*_group×time_=0.01), but only in April (∼3-fold higher). Levels of *ucn2a* in both the middle intestine (Fig. 3E; *P*_group_=0.01, *P*_time_=0.13, *P*_group×time_=0.67) and posterior intestine (Fig. 3F; *P*_group_<0.001, *P*_time_=0.64, *P*_group×time_=0.95) were 30% and 50% higher overall in smolts, respectively. In the middle intestine, abundance of *ucn2b* (Fig. 3G; *P*_group_=0.30, *P*_time_=0.007, *P*_group×time_=0.06) was ∼45% lower through April and May when comparing averaged values combining both groups. In contrast, levels of *ucn2b* in the posterior intestine (Fig. 3H; *P*_group_=0.23, *P*_time_=0.13, *P*_group×time_<0.001) were ∼4.5-fold higher in parr than smolts in July.

**Fig. 4.**
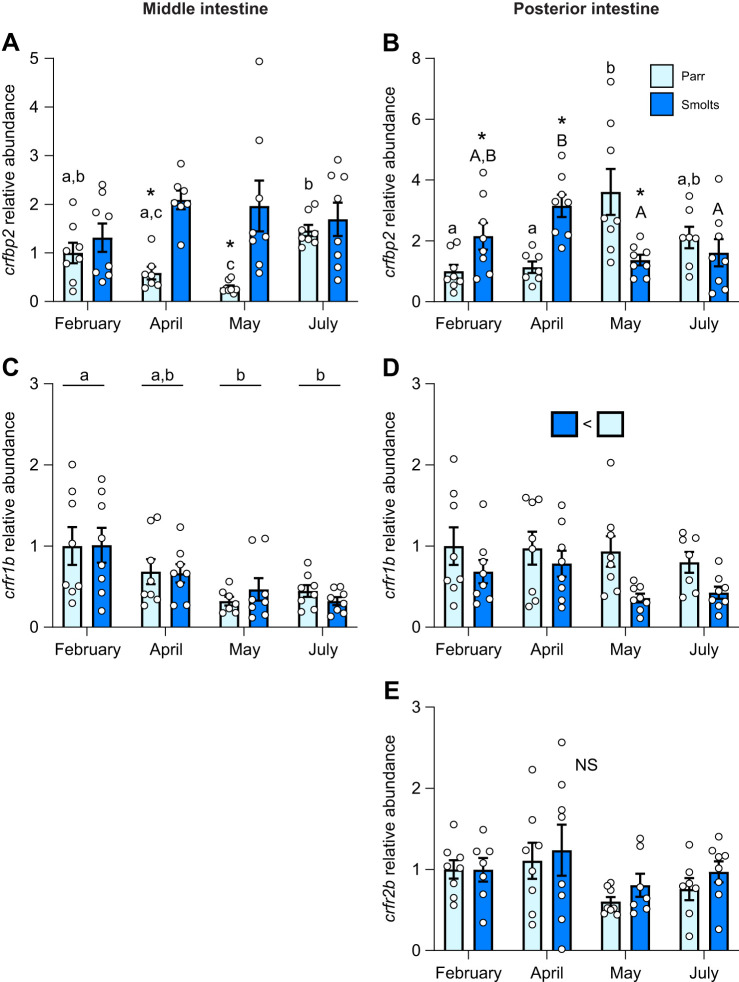
**Changes in intestinal CRF binding proteins and receptors during smoltification.** Seasonal changes in transcript abundance of CRF binding protein 2 (*crfbp2*; A,B), CRF receptor 1b (*crfr1b*; C,D) and CRF receptor 2b (*crfr2b*; E) in the middle (left) or posterior (right) region of the intestine in pre-migratory parr and migratory smolt Atlantic salmon. Significant differences (*P*<0.05; two-way ANOVA) are depicted using either letters (across time; uppercase=within smolts, lowercase=within parr; underlined=overall time effect), filled oversized squares (between groups across all time points) or asterisks (between groups within a time point). Data are expressed relative to parr in February and have been normalized to the geometric mean of *ef1a* and *rpl13a*. Values are represented as means±s.e.m. and individual data points are shown (*N*=5–8). NS, no significant differences.

The abundance of *crfbp2* in the middle intestine (Fig. 4A; *P*_group_<0.001, *P*_time_=0.004, *P*_group×time_<0.001) of parr decreased 40–70% in April through May such that levels in smolts were up to ∼6.5-fold higher than levels in parr during this period. Levels of *crfbp2* in the posterior intestine (Fig. 4B; *P*_group_<0.001, *P*_time_=0.004, *P*_group×time_<0.001) were also 2- to 3-fold higher in smolts in February and April. However, levels of *crfbp2* in parr increased 3-fold in May resulting in parr having ∼2.5-fold higher levels than smolts during peak smoltification. Levels of *crfr1b* in the middle intestine (Fig. 4C; *P*_group_=0.84, *P*_time_<0.001, *P*_group×time_=0.74) decreased by 60% across time when comparing averaged values combining both groups, whereas levels of *crfr1b* in the posterior intestine (Fig. 4D; *P*_group_=0.002, *P*_time_=0.25, *P*_group×time_=0.49) were ∼40% lower in smolts versus parr across all sampling points. No differences in *crfr2b* levels were detected in the posterior intestine (Fig. 4E; *P*_group_=0.42, *P*_time_=0.21, *P*_group×time_=0.86) and *crfr2b* did not consistently amplify in the middle intestine.

As previously reported (Culbert et al., 2022, 2025a), plasma osmolality (Table S2) was slightly higher (∼2%) in smolts versus parr through the spring and summer. Plasma cortisol values (Table S2) were also ∼4.7× higher in smolts than parr and were 4- to 8-fold higher overall in April than in Feb, May or July.

### Effects of FW–SW transfer on the intestine CRF system (parr and smolts)

**Fig. 5.**
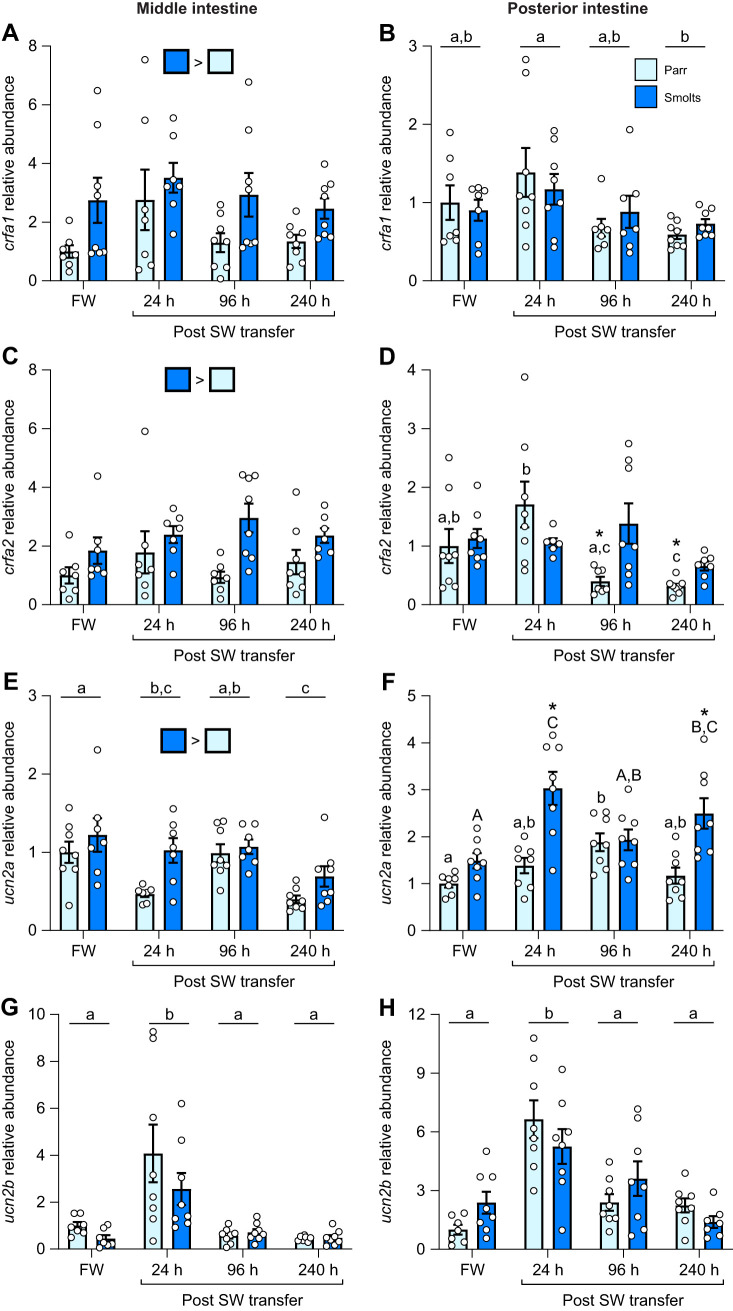
**Effects of seawater (SW) transfer on intestinal CRF ligands.** Changes in transcript abundance of *crfa1* (A,B), *crfa2* (C,D), *ucn2a* (E,F) and *ucn2b* (G,H) in the middle (left) or posterior (right) region of the intestine in pre-migratory parr and migratory smolt Atlantic salmon during peak smoltification in May. Significant differences (*P*<0.05; two-way ANOVA) are depicted using either letters (across time; uppercase=within smolts, lowercase=within parr; underlined=overall time effect), filled oversized squares (between groups across all time points) or asterisks (between groups within a time point). Data are expressed relative to parr in fresh water (FW) and have been normalized to the geometric mean of *ef1a* and *rpl13a*. Values are represented as means±s.e.m. and individual data points are shown (*N*=5–8).

Levels of *crfa1* in the middle intestine (Fig. 5A; *P*_group_<0.001, *P*_time_=0.23, *P*_group×time_=0.91) were not significantly affected by SW transfer, but levels were ∼80% higher in smolts across all sampling points. In the posterior intestine, levels of *crfa1* (Fig. 5B; *P*_group_=0.65, *P*_time_=0.01, *P*_group×time_=0.72) declined as salmon acclimated to SW when comparing averaged values combining both groups. Like *crfa1*, levels of *crfa2* in the middle intestine (Fig. 5C; *P*_group_<0.001, *P*_time_=0.38, *P*_group×time_=0.44) were also ∼85% higher in smolts versus parr overall. Levels of *crfa2* in the posterior intestine (Fig. 5D; *P*_group_=0.002, *P*_time_<0.001, *P*_group×time_=0.009) also decreased across time, especially in parr. Levels of *ucn2a* in the middle intestine (Fig. 5E; *P*_group_=0.002, *P*_time_<0.001, *P*_group×time_=0.32) were ∼40% higher in smolts than parr and averaged values combining both groups decreased by ∼50% during SW acclimation. In contrast, levels of *ucn2a* in the posterior intestine (Fig. 5F; *P*_group_<0.001, *P*_time_=0.001, *P*_group×time_=0.004) increased ∼2-fold in both groups but were twice as high in smolts than parr at 24 h and 240 h post-transfer. Levels of *ucn2b* in both the middle (Fig. 5G; *P*_group_=0.15, *P*_time_<0.001, *P*_group×time_=0.09) and posterior intestine (Fig. 5H; *P*_group_=0.72, *P*_time_<0.001, *P*_group×time_=0.053) transiently increased ∼3- to 6-fold 24 h after SW transfer when comparing averaged values combining both groups.

**Fig. 6.**
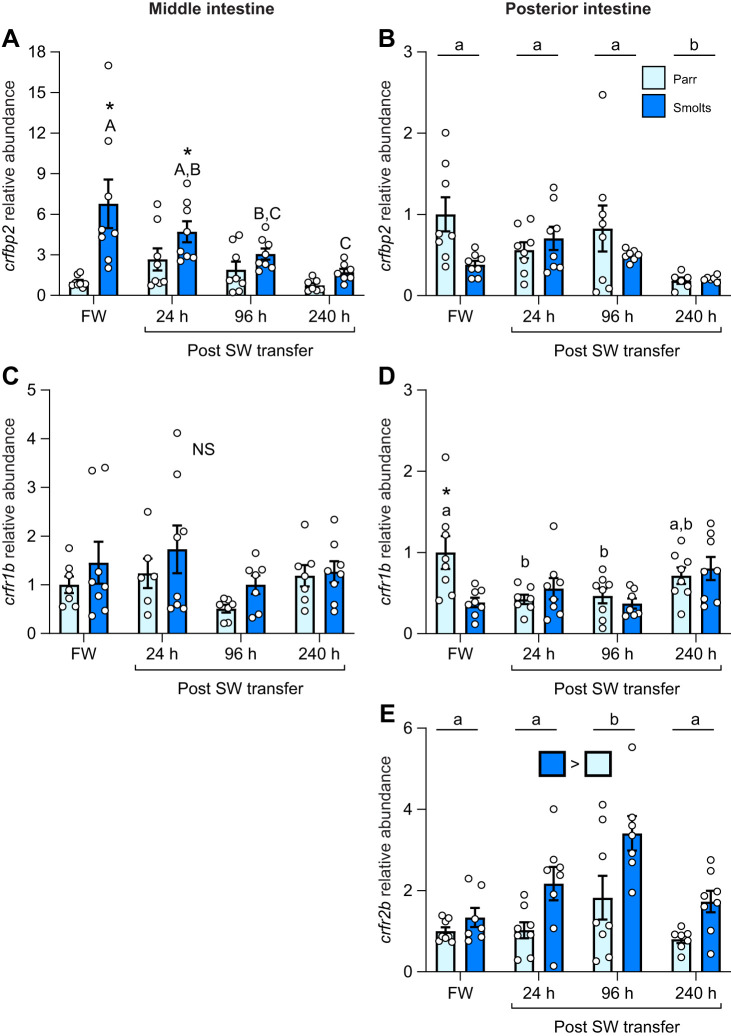
**Effects of seawater (SW) transfer on intestinal CRF binding proteins and receptors.** Changes in transcript abundance of *crfbp2* (A,B), *crfr1b* (C,D) and *crfr2b* (E) in the middle (left) or posterior (right) region of the intestine in pre-migratory parr and migratory smolt Atlantic salmon. Significant differences (*P*<0.05; two-way ANOVA) are depicted using either letters (across time; uppercase=within smolts, lowercase=within parr; underlined=overall time effect), filled oversized squares (between groups across all time points) or asterisks (between groups within a time point). Data are expressed relative to parr in fresh water (FW) and have been normalized to the geometric mean of *ef1a* and *rpl13a*. Values are represented as means±s.e.m. and individual data points are shown (*N*=5–8). NS, no significant differences.

Levels of *crfbp2* in the middle intestine (Fig. 6A; *P*_group_<0.001, *P*_time_=0.001, *P*_group×time_=0.03) were initially ∼7-fold higher in smolts versus parr, but levels declined by ∼75% as smolts acclimated to SW. Levels of *crfbp2* in the posterior intestine (Fig. 6B; *P*_group_=0.08, *P*_time_=0.01, *P*_group×time_=0.06) were not different between parr and smolts but decreased by ∼70% during SW acclimation when comparing averaged values combining both groups. No significant differences were detected for *crfr1b* in the middle intestine (Fig. 6C; *P*_group_=0.13, *P*_time_=0.07, *P*_group×time_=0.66). While levels of *crfr1b* in the posterior intestine (Fig. 6D; *P*_group_=0.15, *P*_time_=0.01, *P*_group×time_=0.02) were initially 2.6-fold higher in parr versus smolts, transcript abundance declined to levels consistent with smolts by 24 h post-transfer. Levels of *crfr2b* in the posterior intestine (Fig. 6E; *P*_group_<0.001, *P*_time_=0.002, *P*_group×time_=0.38) were ∼80% higher in smolts than parr and were ∼2-fold higher at 96 h post-transfer when comparing averaged values combining both groups. However, *crfr2b* did not consistently amplify in the middle intestine.

As previously reported (Culbert et al., 2022, 2025a), plasma osmolality (Table S2) in parr increased by ∼50% 24 h after SW transfer and remained ∼10% higher after 96 or 240 h, while plasma osmolality in smolts was only minimally affected by SW transfer. Plasma cortisol levels (Table S2) increased markedly (340-fold) in parr 24 h after SW transfer, while plasma cortisol levels in smolts were elevated to a lesser degree after 24 h (14-fold) and 240 h (38-fold).

### Effects of SW–FW transfer on the intestine CRF system

**Fig. 7.**
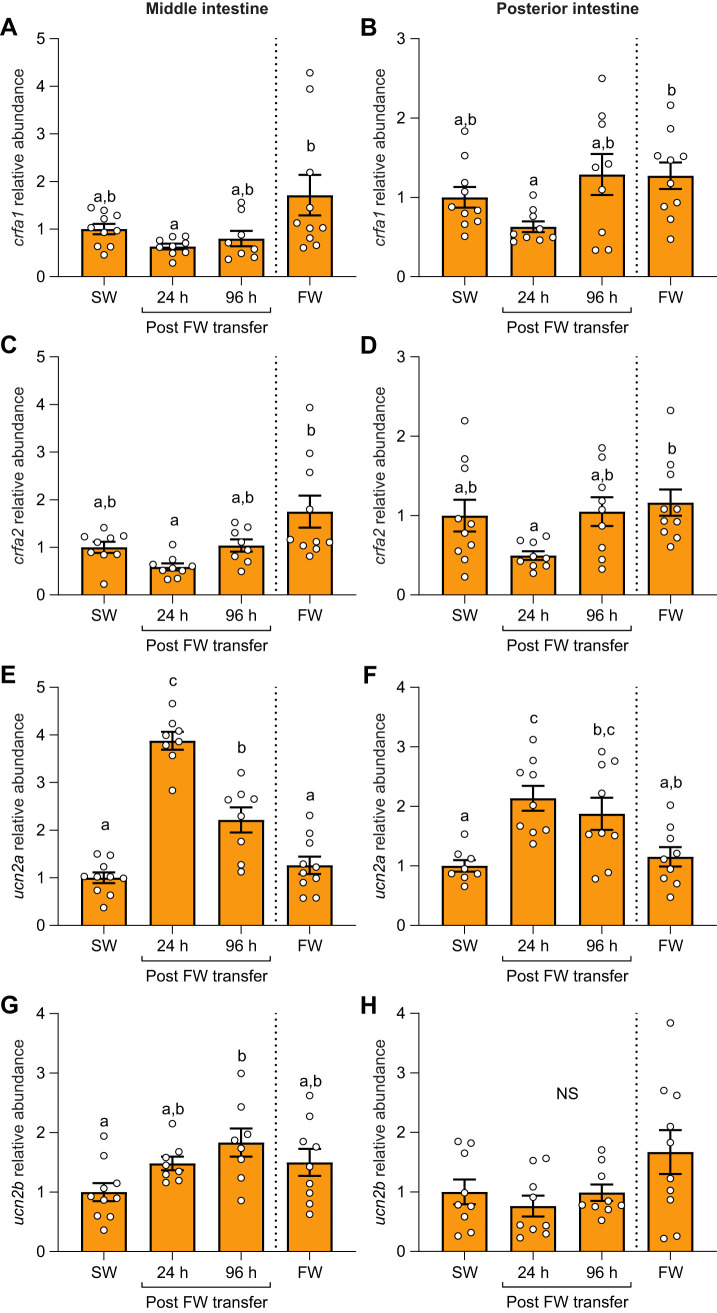
**Effects of SW to freshwater (FW) transfer on CRF ligands.** Changes in transcript abundance of *crfa1* (A,B), *crfa2* (C,D), *ucn2a* (E,F) and *ucn2b* (G,H) in the middle (left) and posterior (right) regions of the intestine in Atlantic salmon post-smolts. Significant differences (*P*<0.05; one-way ANOVA) are depicted using letters. Data are expressed relative to SW-acclimated fish and have been normalized to the geometric mean of *ef1a* and *rpl13a*. Values are represented as means±s.e.m. and individual data points are shown (*N*=7–10). The FW group was never transferred to seawater (indicated using the dashed line). NS, no significant differences.

Levels of *crfa1* in the middle (Fig. 7A; *P*=0.009) and posterior intestine (Fig. 7B; *P*=0.04), as well as *crfa2* in the middle (Fig. 7C; *P*<0.001) and posterior intestine (Fig. 7D; *P*=0.04), transiently decreased by ∼40–50% at 24 h post-transfer. In contrast, levels of *ucn2a* in the middle (Fig. 7E; *P*<0.001) and posterior intestine (Fig. 7F; *P*<0.001) transiently increased 4- and 2-fold, respectively. Levels of *ucn2b* in the middle intestine (Fig. 7G; *P*=0.03) also increased, reaching a peak of ∼70% at 96 h post-transfer, whereas a significant difference was not detected in the posterior intestine (Fig. 7H; *P*=0.26).

**Fig. 8.**
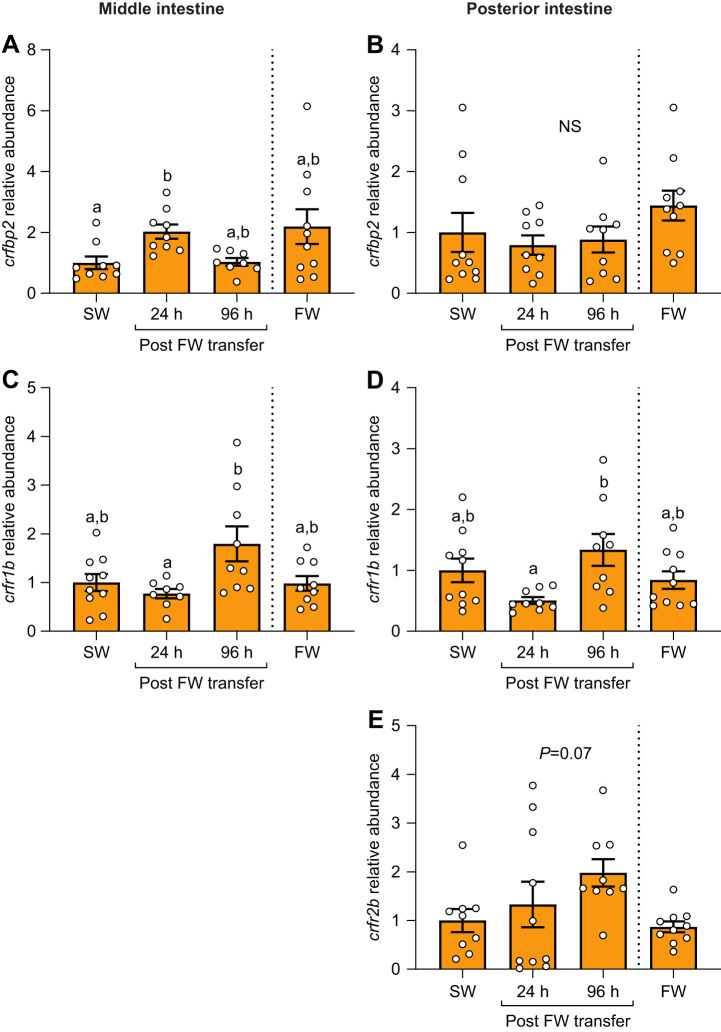
**Effects of SW–FW transfer on intestinal CRF binding proteins and receptors.** Changes in transcript abundance of *crfbp2* (A,B), *crfr1b* (C,D) and *crfr2b* (E) in the middle (left) and posterior (right) regions of the intestine in Atlantic salmon post-smolts. Significant differences (*P*<0.05; one-way ANOVA) are depicted using letters. Data are expressed relative to SW-acclimated fish and have been normalized to the geometric mean of *ef1a* and *rpl13a*. The *P*-value is reported in F because this result trended towards significance (*P*<0.10). Values are represented as means±s.e.m. and individual data points are shown (*N*=7–10). The FW group was never transferred to seawater (indicated using the dashed line). NS, no significant differences.

Levels of *crfbp2* were transiently upregulated 2-fold at 24 h post-transfer in the middle intestine (Fig. 8A; *P*=0.02), but no differences were detected in the posterior intestine (Fig. 8B; *P*=0.19). Similarly, levels of *crfr1b* in the middle (Fig. 8C; *P*=0.02) and the posterior intestine (Fig. 8D; *P*=0.02) were transiently elevated at 96 h post-transfer. Levels of *crfr2b* in the posterior intestine (Fig. 8E; *P*=0.07) exhibited a non-significant tendency to be higher at 96 h post-transfer, but did not consistently amplify in the middle intestine.

As previously reported (Culbert et al., 2022, 2025a), plasma osmolality values (Table S2) decreased by ∼1–2% after transfer from SW to FW, while plasma cortisol levels (Table S2) were ∼70% lower 96 h post-transfer.

## DISCUSSION

The CRF system is an important regulator of ion and water transport in the mammalian intestine (Rodiño-Janeiro et al., 2015; Stengel and Taché, 2009), but whether it contributes to osmoregulatory processes in the intestine of teleost fishes is not clear. Our *in vitro* results indicate that the CRF system has osmoregulatory functions in the intestine of Atlantic salmon since treatment of intestinal sacs with CRF peptides reduced rates of ion and water absorption. Specifically, these effects appear to be primarily mediated by the activation of CRFR2 since inhibition of CRFR1 had no impact on the ability of CRF peptides to reduce Na^+^ and water uptake. These results contrast to the process in mammals where the osmoregulatory actions of the intestinal CRF system are primarily mediated by CRFR1 (Rodiño-Janeiro et al., 2015). Additionally, in contrast to our prediction that CRF system activity would decrease during smoltification and/or following SW transfer – reflecting a greater need for intestinal water uptake during these periods (Collie and Bern, 1982; Grosell et al., 2007; Kerstetter and White, 1994; Loretz et al., 1982; Shehadeh and Gordon, 1969) – transcript levels of CRF system components in the middle and posterior regions of the intestine were generally elevated in smolts and transiently increased following changes in environmental salinity (either FW–SW or SW–FW). Therefore, despite *in vitro* evidence that the CRF system can reduce rates of intestinal ion and water absorption, the physiological conditions under which the intestinal CRF system contributes to osmoregulation remain to be determined.

**Fig. 9.**
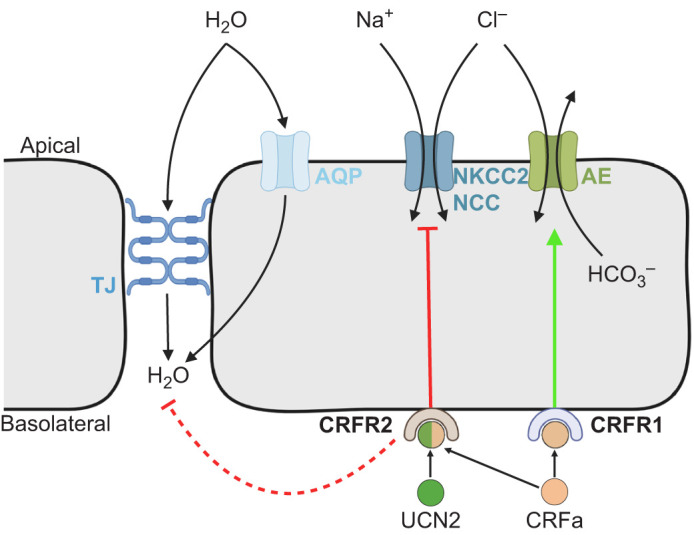
**Proposed osmoregulatory actions of the corticotropin-releasing factor (CRF) system in the intestine.** Activation of CRFR2 indirectly (dashed lines) reduces water absorption – either intracellularly via aquaporins (AQP) or transcellularly via tight junctions (TJ) – by decreasing uptake of luminal Na^+^ and Cl^−^ via apical Na^+^-K^+^-2Cl^−^ type 2 (NKCC2) and/or the Na^+^-Cl^−^ (NCC) cotransporters. Activation of CRFR1 increases rates of luminal Cl^−^ uptake by stimulating apical activity of a nHCO_3_^−^/Cl^−^ anion exchanger (AE). Note that while CRFa and UCN2 are depicted in the diagram in accordance with our experimental design, several CRF peptides likely contribute *in vivo*. Green arrow and red line indicate stimulatory and inhibitory effects of CRFRs, respectively. Created in BioRender. Bernier, N. (2025) https://BioRender.com/h61u502

As in other epithelial tissues, water uptake across the intestine is primarily driven by the uptake of ions (reducing luminal osmolality) and the accumulation of ions in the lateral intercellular space adjacent to the basolateral membrane of epithelial cells, which creates a favorable osmotic gradient for water absorption (Larsen et al., 2009; Whittamore, 2012). However, since the majority of Na^+^ and Cl^−^ that is absorbed across the apical surface of the intestine occurs via cotransporters – either NKCC2 or the Na^+^–Cl^−^ cotransporter (NCC) – rates of Na^+^, Cl^−^ and water transport all often follow the same pattern (Grosell, 2011a; Whittamore, 2012). Indeed, the osmoregulatory effects associated with the CRF system in mammals and insects involve changes in NKCC1 activity (Coast, 2007; Liu et al., 2021). As such, the parallel reductions in Na^+^, Cl^−^ and water uptake following UCN2 treatment (i.e. activation of CRFR2) suggest that changes in NKCC2 activity may be involved. In contrast, rates of Cl^−^ transport were not reduced following treatment with CRFa (i.e. activation of CRFR1 and CRFR2). In addition to cotransport with Na^+^ via NKCC2 and NCC, smaller amounts of Cl^−^ are also absorbed across the apical surface of the intestine via a nHCO_3_^−^/Cl^−^ exchanger. This transporter is generally more active in SW-acclimated fish – as indicated by higher rates of HCO_3_^−^ secretion into the intestine (Gilmour et al., 2012; Grosell et al., 2007; Ruiz-Jarabo et al., 2017) – but intestinal HCO_3_^−^ secretion by the anterior intestine also plays an important role in neutralizing acidic chyme coming from the stomach during digestion in FW-acclimated fish (Bucking and Wood, 2008; Cooper and Wilson, 2008; Taylor et al., 2007; Wood and Eom, 2019). Furthermore, previous studies have suggested that an apical nHCO_3_^−^/Cl^−^ exchanger may even be a significant contributor to Cl^−^ uptake in some FW fish (Scott et al., 2006; Wood et al., 2010). As such, the different effects of CRFa versus UCN2 treatment on rates of Cl^−^ transport could be mediated by differential effects on an apical nHCO_3_^−^/Cl^−^ exchanger. Specifically, activation of CRFR1 may stimulate rates of Cl^−^ uptake via activation of this exchanger. Indeed, when the activity of CRFR1 was blocked using the CRFR1-specific antagonist antalarmin, the effects of CRFa on Cl^−^ transport were reduced, mimicking the effects observed with UCN2. Transcript levels of *crfr2b* are ∼50-fold higher in the posterior intestine compared with the middle intestine, suggesting that the actions of CRFR2 occur primarily in the posterior intestine – although, future studies evaluating potential region-specific, post-transcriptional differences in the intestine are needed. In contrast, levels of *crfr1b* were ∼4-fold lower in the posterior versus middle intestine, which may reflect lower rates of HCO_3_^−^ secretion in the posterior intestine (Grosell, 2011b; Wilson et al., 1996). Since our *in vitro* preparations included the entire intestine posterior to the caeca, it is possible that the dual activation of both CRFRs caused region-specific responses via different receptor subtypes. We propose that activation of CRFR2 in the posterior intestine reduces rates of water absorption by suppressing activity of apical NCC and/or NKCC2, whereas activation of CRFR1 in the anterior intestine potentially stimulates rates of Cl^−^ uptake via increased activity of an apical nHCO_3_^−^/Cl^−^ exchanger (Fig. 9). However, this hypothetical mechanism still needs to be explicitly tested. Curiously, Mainoya and Bern (1982) found that rates of Cl^−^, Na^+^ and water transport were all reduced when the anterior intestine of Mozambique tilapia was treated with UTS1 – which also activates both CRFR subtypes (Arai et al., 2001; Manuel et al., 2014; Pohl et al., 2001) – suggesting potential species-specific distribution and/or effects of CRFR1 versus CRFR2 in the teleost intestine. Clearly, further experiments are needed to determine the mechanism(s) underlying the effects observed in the current study, and whether they are conserved across other teleosts.

While our *in vitro* data are consistent with the CRF system having osmoregulatory functions in teleost fishes – as in mammals and insects (Cannell et al., 2016; Stengel and Taché, 2009) – it is not clear whether the transcriptional changes in the intestinal CRF system observed during smoltification or following changes in environmental salinity are directly related to these osmoregulatory functions. In general, rates of intestinal water absorption are greater in smolts than in parr (Collie and Bern, 1982; Kerstetter and White, 1994; Loretz et al., 1982), which is consistent with the greater osmoregulatory capacity of smolts in SW. If the CRF system is contributing to seasonal changes in water absorption in the intestine of smolts, then intestine CRF system activity would be expected to be lower in smolts than in parr. Indeed, smolts had higher levels of *crfbp2* in the middle intestine and lower levels of *crfr1b* in the posterior intestine – and these differences were greatest during peak smoltification in May – which is consistent with reduced CRF system activity and, potentially, an enhanced capacity for water absorption. However, we also found that transcript levels of *crfa1*, *crfa2* and *ucn2a* were higher in smolts versus parr throughout the spring, indicating greater CRF system activity and a reduced capacity for water absorption across the intestine. Similarly, transcript levels of CRF ligands in the caudal neurosecretory system (CNSS) – the primary source of circulating CRF ligands in fish – are higher in smolts (Culbert et al., 2025b), which may also contribute to increased activation of the intestinal CRF system. It is difficult to explain these contrasting changes in transcript levels of different CRF system components, but a better understanding of how other hormone systems influence CRF system regulation would be helpful. Regardless, increased transcription of CRF ligands during smoltification appears to be a widespread response across tissues in Atlantic salmon. Levels of *crfa1*, *crfa2* and *crfb2* in the gills (Culbert et al., 2025a), *uts1a* in the hypothalamus and *crfb1* in the preoptic area of the brain (Culbert et al., 2022), and *uts1a*, *uts1b* and *crfb1* in the CNSS (Culbert et al., 2025b) were all elevated in smolts versus parr during the spring. Therefore, while it is likely that transcriptional changes in levels of CRF ligands within each of these tissues are associated with a different collection of functional outcomes, activation of the CRF system is clearly a widespread response during smoltification.

Salmon begin to drink large amounts of SW within 6 h following SW transfer (Fuentes et al., 1996; Usher et al., 1988) and absorption of this water by the gastrointestinal tract is critical to avoid dehydration. Curiously, the transcriptional changes observed in the intestinal CRF system following SW transfer (reduced abundance of *crfbp2* combined with transient increases in *ucn2a*, *ucn2b* and *crfr2b* levels) suggest that CRF system activity increases (and therefore might reduce rates of intestinal water absorption) in the initial days following SW transfer. Additionally, we observed similar transient increases in *ucn2a* and *ucn2b* levels – as well as a non-statistically significant rise in *crfr2b* abundance in the posterior intestine – when SW-acclimated fish were transferred to FW. While activation of the CRF system following transfer from SW to FW could serve to reduce water absorption as the need for drinking subsides in a hypoosmotic environment, potential osmoregulatory benefits associated with CRF-mediated reductions in water absorption following SW transfer are less clear. Indeed, *in vivo* rates of water absorption across the intestine increase markedly following SW transfer (Grosell et al., 2007; Shehadeh and Gordon, 1969) and the osmoregulatory effects of UTS1 on the intestine were stronger in FW- versus SW-acclimated tilapia (Mainoya and Bern, 1982). However, like our results for the CRF system, Ruhr et al. (2015) also reported transcriptional activation of the guanylin system – which reduces rates of water and ion absorption in teleosts (Ruhr et al., 2014; Takei and Yuge, 2007; Yuge and Takei, 2007) – in the posterior intestine during acclimation of marine Gulf toadfish (*Opsanus beta*) to hypersaline SW (60 ppt). Functionally, Ruhr et al. (2014) suggest that guanylin-mediated reductions in water absorption may help with the elimination of CaCO_3_ precipitates in the intestine, which accumulate in large amounts in the intestine of SW-acclimated fish (Grosell, 2006, 2011b). Thus, the CRF system may also be activated during SW acclimation to assist with similar osmoregulatory functions in the intestine, although confirmation that the observed transcriptional changes lead to translational (or other post-transcriptional) responses is still needed.

Alternatively, it is possible that the transient activation of the CRF system following changes in environmental salinity is associated with other functional changes occurring in the intestine during this period. For example, FW and SW contain different amounts and types of pathogens (Dehler et al., 2017; Lokesh and Kiron, 2016; Nekouei et al., 2019) and many immunological changes occur following SW transfer of Atlantic salmon (Johansson et al., 2016; Wang et al., 2020). We previously found that activation of the CRF system causes transcriptional changes in immune-related genes in the gills of Atlantic salmon (Culbert et al., 2025a) and the osmoregulatory actions of CRF in the posterior intestine of rodents are thought to help flush out pathogens (Stengel and Taché, 2009). Therefore, activation of the CRF system during the initial period following changes in environmental salinity (either FW–SW or SW–FW) may help fish combat novel pathogens prior to acquiring more specific microbial and/or immunological defense mechanisms. Future studies should directly evaluate immune-related responses of the intestinal CRF system and, in addition, measure changes in protein levels of the major CRF system components in the intestine to better understand the functional consequences of the transcriptional changes observed in the current study.

More broadly, our results also indicate that paralogs of some components of the CRF system (e.g. CRFBP and CRFR2) – but perhaps not others (e.g. CRFa, UCN2 and CRFR1) – have undergone sub-functionalization within the intestine, as indicated by differences in transcript abundance. Interestingly, we also observed strong paralog-specific transcription patterns for several CRF system component paralogs in Atlantic salmon gills (Culbert et al., 2025a), although a different collection of genes was identified (e.g. CRFb, UCN2 and CRFR2). However, both the intestine and gills exhibit high levels of CRFa, UCN2 and UCN3 (Culbert et al., 2025a; current study), suggesting that these peptides have paracrine/autocrine roles in both tissues. In contrast, CRFb and UTS1 are the major ligands produced by the caudal neurosecretory system in salmonids (Bernier et al., 2008; Craig et al., 2005; Culbert et al., 2025b), which is thought to be the primary endocrine source of CRF signaling in teleosts. Therefore, it appears that different sets of ligands within the salmonid CRF system are responsible for endocrine (CRFb and UTS1) versus paracrine/autocrine (CRFa, UCN2 and UCN3) regulation of peripheral tissues. However, additional studies are required to determine whether these patterns are consistent in other peripheral tissues and other species.

In conclusion, we have shown that the activity of the CRF system can directly influence the transport of ions and water across the intestine of Atlantic salmon. We have also shown that transcript levels of the major components of the intestinal CRF system change during smoltification and following changes in environmental salinity, further supporting a potential osmoregulatory role for the CRF system. However, there is still much to learn about the osmoregulatory contributions of this hormone system. For instance, future studies employing region-specific intestinal sacs – including the anterior portion of the intestine, which was not assessed in the current study – combined with pharmacological tools (e.g. NKCC2 and/or NCC inhibitors) will be necessary to determine the mechanisms by which the CRF system influences rates of ion and water movement across the intestine. Additionally, the efficacy and specificity of antagonists for CRFR2 (none of which have been validated in fish) should be established to confirm the apparent actions of CRFR2 in the intestine. More generally, increasing the realism of environmental conditions (i.e. those better mimicking the *in vivo* environment; Bucking et al., 2024) during future *in vitro* experiments (e.g. pH, [ammonia], [ions], *P*_CO_2__ and *P*_O_2__ inside intestinal sacs), as well as manipulating CRF system activity *in vivo*, will provide further insight into the potential region- and/or context-dependent contributions of the intestinal CRF system. Nonetheless, the results of the current study, combined with those of Mainoya and Bern (1982), indicate that the intestinal CRF system of teleosts has direct osmoregulatory actions, consistent with those observed in the mammalian intestine and Malpighian tubules of insects. Overall, our results support and extend the evolutionary breadth of the CRF system's osmoregulatory actions.

## Supplementary Material

10.1242/jexbio.250052_sup1 Supplementary information
