# Supplementary material for: Osmoregulatory contributions of the corticotropin-releasing factor system in the intestine of Atlantic salmon
Source: J Exp Biol. 2025 May 15;228(14):jeb250052. doi: 10.1242/jeb.250052 (PMC12091946; doi:10.1242/jeb.250052)
Supplement: Supplementary information [file jexbio-228-250052-s1.pdf]

Table S1. Gene specific primers used for real-time polymerase chain reaction (qPCR).

	Primer Sequence (5' to 3')	Amplicon Size (bp)	Efficiency (%)	Accession Number	Reference
crfa1	F: TCGCCGAACACATCTCCTG<br>R: TGCGTGAGCTGAAGTTGTAA	74	101	XM_014139988	Culbert et al., 2022
crfa2	F: GGTCAACAGGGCTTTACTTCA<br>R: AACCGATTGCTGTTACCGAC	69	103	XM_014190344	Culbert et al., 2022
crfb1	F: CTTGATCCATCACTCGTGGAAA<br>R: GTCAGGGGTTCAACGAGATC	98	108	XM_014181363	Culbert et al., 2022
crfb2	F: GAGGAAGGCAGCTCTCAACT<br>R: TCATGTCGGGATCAACAGGAA	84	96	XM_014159556	Culbert et al., 2022
crfbp1	F: GTTTCTGAAGGGAGACACCAGA<br>R: TCGCCTGTAAATGAATGTCTCG	118	107	XM_014128333	Culbert et al., 2025
crfbp2	F: GTGAGGGTGTTAGCAGGGTG<br>R: CCACAGTCCCAAAACGACAC	116	103	NM_001173799	Culbert et al., 2025
crfr1a	F: AGCTACAGAGGCTGACAATGG<br>R: CTGTGCTGCCTTTGGCAAAG	69	99	XM_014204418	Culbert et al., 2022
crfr1b	F: CAGAGGGTCAGGATGACAATG<br>R: GTGGCAAAAAGTCAGCTCTTG	59	108	XM_014192620	Culbert et al., 2022
crfr2a	F: ACCTGGATCGCCAAGTTCA<br>R: AGTGCTCTGTCTCTGTCTTCA	141	107	XM_014140566	Culbert et al., 2025
crfr2b	F: GACCAAATCCAAAGGTCACCT<br>R: GCTCTCTGTCCTTATGCAGC	123	99	XM_014191256	Culbert et al., 2025
ef1α	F: TCCTGCGGAGTCTCAAAACC<br>R: CGTTGGGTTCTTTTCCTGCG	96	103	XM_014141923	Culbert et al., 2022
rpl13a	F: GGACAAGCTGCACTGGAGAG<br>R: GTGGGCTTCAGACGGACAAT	113	93	XM_014128281	Culbert et al., 2025
ucn2a	F: CATGCGTCTGTGTCTGAGC<br>R: AGCCAACAGGTCACTTTCTG	115	93	ENSSSAT00000205481	Culbert et al., 2025
ucn2b	F: GCAACCAGTGCCAGATAACG<br>R: GGGTGTTGGATGGAATTCTGG	137	109	XM_014133639	Culbert et al., 2025

Journal of Experimental Biology • Supplementary information

ucn3	F: CGCAGATTGGACGAAGGAAG<br>R: ATGGATAATCAGGGTAGCGGC	71	100	XM_045712114<br>XM_014185247	Culbert et al., 2025
uts1a	F: CAGTGTCTGTAGACCACGG<br>R: TATCACCAGCCTTCAGCAAC	89	104	XM_014205273	Culbert et al., 2022
uts1b	F: GCAGTCTACTACAATCGCCAT<br>R: AAACGGTGCCTTGATCGC	103	101	XM_014144548	Culbert et al., 2022

crf, corticotropin-releasing factor; *crfbp*, corticotropin-releasing factor binding protein; *crfr*, corticotropin-releasing factor receptor; *ef1α*, elongation factor 1α; *rpl13a*, ribosomal protein L13a; *ucn*, urocortin; *uts*, urotensin.

Journal of Experimental Biology • Supplementary information

Table S2. Plasma cortisol (ng mL^{-1}) and osmolality (mOsm kg^{-1}) values for Atlantic salmon (*Salmo salar*) from experiments 1, 2, and 4. Values are presented as means ± SEM. Significant differences are indicated in **bold.** Small letters indicate differences across time within a group, asterisks indicate a difference between groups within a timepoint, and capital letters indicate an overall difference between groups or across times based on post hoc analysis. Note that these data have previously been reported in Culbert et al. (2022 & 2025).

Variable	Exp		Sampling Time				P-value	
Plasma Cortisol	2		Feb^{Y}	April^{Z}	May^{Y}	July^{Y}		
		Parr^{A}	0.2 ± 0.1	1.8 ± 0.4	0.5 ± 0.1	0.2 ± 0.1	**Time**	**<0.001**
		Smolts^{B}	0.8 ± 0.2	7.2 ± 2.9	2.4 ± 0.6	2.2 ± 0.7	**Group**	**<0.001**
							Interaction	0.34
			FW	24h	96h	240h		
		Parr	0.5 ± 0.1^{a*}	170.5 ± 25.1^{b*}	18.0 ± 7.8^{c}	12.7 ± 4.2^{c*}	**Time**	**<0.001**
		Smolts	2.4 ± 0.6^{y}	35.2 ± 8.6^{z}	6.2 ± 1.4^{y}	90.7 ± 25.4^{z}	Group	0.15
							**Interaction**	**<0.001**
	3		SW	24h	96h	FW		
			2.0 ± 0.8^{a}	2.4 ± 0.8^{a}	0.6 ± 0.5^{b}	2.9 ± 1.1^{a}	**Time**	**<0.001**
Plasma Osmolality	2		Feb	April	May	July		
		Parr^{A}	309 ± 5	304 ± 6	302 ± 4	310 ± 4	Time	0.20
		Smolts^{B}	315 ± 4	304 ± 5	320 ± 3	312 ± 3	**Group**	**0.04**
							Interaction	0.14
			FW	24h	96h	240h		
		Parr	302 ± 4^{a*}	456 ± 11^{c*}	342 ± 9^{b}	326 ± 6^{b*}	**Time**	**<0.001**
		Smolts	320 ± 3^{yz}	327 ± 3^{z}	317 ± 4^{yz}	313 ± 2^{y}	Group	0.06
							**Interaction**	**<0.001**
	3		SW	24h	96h	FW		
			307 ± 1^{b}	301 ± 1^{a}	303 ± 1^{a}	303 ± 1^{a}	**Group**	**0.001**

References

Culbert, B. M., Regish, A. M., Hall, D. J., McCormick, S. D. and Bernier, N. J. (2022). Neuroendocrine regulation of plasma cortisol levels during smoltification and seawater acclimation of Atlantic salmon. *Front. Endocrinol.* **13**, 859817.

Culbert, B. M., Mossington, E., McCormick, S. D. and Bernier, N. J. (2025). Regulation and function of the gill corticotropin-releasing factor system during osmoregulatory disturbances in Atlantic salmon. *J. Exp. Biol.* **228**, jeb.248168.

Journal of Experimental Biology • Supplementary information
